# Cathepsin B hyperactivation facilitates exosome release of CVB3 particles and exacerbation of acute pancreatitis by impairing lysosomal integrity and acidification

**DOI:** 10.1128/mbio.03111-25

**Published:** 2025-11-24

**Authors:** Tianming Liang, Zhipeng Zhang, Le Xu, Zhirong Sun, Wei Xu

**Affiliations:** 1 Jiangsu Provincial Key Laboratory of Infection and Immunity, Soochow University Institutes of Biology and Medical Sciences 625756, Suzhou, China; The University of Iowa, Iowa City, Iowa, USA

**Keywords:** acute pancreatitis, CVB3, CTSB, LMP, exosome

## Abstract

Lysosomal cathepsin B (CTSB) exhibits diverse roles in physiological and pathological processes. Upregulation and trypsinogen-activating function of CTSB have been reported in experimental secretagogue-elicited AP. Whether CTSB regulates the lysosome pathway and viral release in viral acute pancreatitis (AP) remains obscure. In a murine model of Coxsackievirus 3 (CVB3)-induced AP, we tested effects of CTSB on lysosome integrity and exosome secretion. CVB3 infection does- and time-dependently upregulates expression and activity of CTSB, the most elevated CTS in pancreas, and induces lysosomal instability and extra-lysosomal translocation of CTSB. Overexpression of CTSB increases viral replication. Inhibition of CTSB with CA074Me reduces virion release via rescuing lysosome integrity and acidity. Mechanically, CTSB hyperactivation and inappropriate cytoplasmic translocation increase viral infections by decreasing LAMP-1 + lysosomes, exacerbating lysosomal membrane permeabilization (LMP), and enhancing exosomal release of virions. Pharmaceutical inhibition of CTSB improves AP pathology via reducing viral infection. Our study reveals a critical role of hyperactivated CTSB in disrupting lysosome integrity, facilitating exosomal release of CVB3 particles and exacerbation of pancreas pathology in AP. These findings offer new insights into the pathogenesis of viral AP and suggest that CTSB and exosome are potential therapeutic targets for viral AP.

**IMPORTANCE:**

This study uncovers a critical role of lysosome cathepsin B (CTSB) in exacerbating viral acute pancreatitis (AP). CVB3 infection of acinar cells induces lysosomal membrane permeability and CTSB cytosolic translocation. Hyperactivated CTSB increases viral infections by decreasing lysosomes, exacerbating LMP, and enhancing exosomal release of virions. Pharmaceutical inhibition of CTSB protects mice against viral dissemination and AP pathology, suggesting that CTSB and exosome are potential therapeutic targets for viral AP.

## INTRODUCTION

Acute pancreatitis (AP) is a sudden inflammatory response of the pancreas, clinically characterized by severe abdominal pain, elevated serum pancreatic enzymes, and pancreatic tissue damage. The disease is primarily characterized by inappropriate activation of trypsinogen, infiltration of inflammatory cells, and destruction of acinar cells (1, 2). The causes and mechanisms of AP remain largely unknown. While alcohol abuse and biliary obstruction are widely recognized as causes of AP, there is a growing concern regarding the increasing cases attributed to infections (3–5). Coxsackievirus B3 (CVB3), a Picornaviridae family member, represents a known viral trigger for AP (6). While primarily associated with viral myocarditis, CVB3 infection induces AP and other clinical manifestations (1, 6–8). The association between CVB3 and AP is increasingly recognized, yet the mechanistic basis involved in viral AP remains poorly characterized. No antiviral treatment exists against CVB3, and no specific or effective treatment of AP is available.

Lysosomes play pivotal roles in macromolecule digestion, autophagy, and cellular homeostasis. Dysfunctional lysosomes, caused by altered acidification, defective enzymes, or compromised membrane integrity, are associated with various pathologies, including cancer, neurodegenerative diseases, inflammatory diseases, and infections (9). CVB3 infection impairs lysosome function by various means (10–12). CVB3 uses proteinase 3C to proteolyze TFEB (transcription factor EB), a master regulator of autophagy and lysosome biogenesis, into a loss-of-function cleavage fragment to disrupt host lysosomal function and enhance viral infection (10). CVB3 protein 2B-ER-insertion initiated calcium outflow and CVB3-induced ROS increase, leading to lysosomal membrane damage (9, 13). Lysosomes are the terminal destination for autophagy. Early endosomes mature into late endosomes or the multivesicular bodies (MVB). CVB3 induces double membrane (DMVs) in the host cells to serve as replication sites. Late in infection, CVB3 increases the formation of late endosomes/MVBs and acidified autophagosomes (amphisomes) in the cytoplasm of host cells, allowing viral particles to mature inside them (14). Lysosome fusion with endosomes/MVBs or autophagosomes, forming highly acidic degradative endolysosomes or autophagolysosomes, is important in degradation and recycling of cellular waste (15). CVB3 evades autophagosomal degradation (by blocking the downstream autophagosome–lysosome fusion steps via cleaving SNAP29) and misuses the endosomal/autophagosomal pathway for viral replication and release (10, 16). Fusion of virion-included MVBs or amphisomes with the plasma membrane leads to non-lytic release of virus-associated exosome into the extracellular environment, increasing intracellular viral dissemination (17).

The exosomes, which originate from the MVB, are 30–150 nm in diameter and are released from the cell upon MVB–plasma membrane fusion, carrying cytoplasmic cargo to the extracellular environment (18). Compromised lysosomal activity leads to elevated exosome release due to disrupted endosome–lysosome fusion processes. Damaged lysosomes may alter molecular contents of exosomes, subsequently modulating their biological functions (15, 19). Alterations in exosome release due to lysosomal dysfunction have been associated with the pathogenesis of Parkinson’s disease (20), alcoholic liver disease (21), and non-alcoholic fatty liver disease (22). Enteroviruses, such as Coxsackievirus and enterovirus 71 (EV-A71), have been revealed to exploit the secretory autophagy–exosome pathway to exit cells (23). Although CVB3 hijacks cellular autophagy for pro-viral functions, the precise mechanisms by which viral proteins or cellular proteins disrupt autophagy or lysosome remain incompletely understood. How lysosomal components modulate the MVB/amphisome formation, membrane fusion, and exosome release remains to be clarified.

Cathepsin B (CTSB) is a powerful lysosomal hydrolase that plays a crucial role in modulating the autophagy process and lysosomal function, participating in various biological processes, including protein degradation, signal transduction, and cell death (24). CTSB is primarily localized within subcellular endosomal and lysosomal compartments. Under homeostatic conditions, CTSB controls lysosomal dynamics and autophagy by cleaving the lysosomal calcium channel MCOLN1, which negatively regulates the efflux of calcium, activating PPP3. This dephosphorylates TFEB and initiates autophagy (24). CTSB was also detected in the cytosol and nuclear fraction of senescent microglial cells, suggesting its extralysosomal function in senescent cell (25). In the initial stage of experimental AP, CTSB plays a major role in pathological trypsinogen activation in the early course of cerulein-induced pancreatitis (26, 27). Cytosol CTSB initiates apoptosis and necrosis of acinar cells in experimental cerulein-induced AP (28). CTSB is upregulated in cardiomyocytes and stimulates the aortic banding-induced activation of TNF-α and cytochrome c release, thereby enhancing pressure overload-induced cardiac hypertrophy and fibrosis (29). CTSB promotes the maturation and secretion of IL-1β and IL-18, thus exacerbating local pancreatic damage (28). Pharmacological inhibition and genetic deletion of CTSB reduce trypsin activation and alleviate the severity of experimental secretagogue-elicited AP (30). During CVB3-induced viral AP, whether hyperactivated and extra-lysosomal located CTSB regulates lysosome function, exosomal viral release, and AP pathology needs further investigation.

In this study, CVB3 infection-induced murine viral AP model was used to investigate the role of cathepsin B (CTSB) in the regulation of lysosome integrity, CVB3 particle exosome release, and viral AP pathology. We identified CTSB as the most significantly upregulated cathepsin family member in the pancreas of CVB3-infected mice. CTSB aberrant activation and inappropriate cytoplasmic translocation led to lysosomal membrane permeabilization (LMP) and dysfunction as well as increased virus-associated exosome release. Inhibition of CTSB mitigated CVB3-induced AP pathology by suppressing CVB3 replication and non-lytic exosomal release. Our findings highlight CTSB as a crucial modulator of lysosome function and exosome release during viral infection, providing a novel target for therapeutic intervention of CVB3-induced AP.

## MATERIALS AND METHODS

### Mice, virus, and cells

Male C57BL/6J mice were obtained from the Soochow University Animals Center and cultured in a pathogen-free environment. All animal experiments were conducted in compliance with the Institutional Animal Care and Use Committee of Soochow University. The study protocols were approved by the Animal Ethical Committee of Soochow University (SYXK2022-0128). CVB3 (Nancy strain) is a gift from Prof. Y. Yang (Key laboratory of viral heart diseases, Zhongshan Hospital, China) was propagated in a HeLa cells monolayer in DMEM (Life Technologies) supplemented with 10% fetal bovine serum (FBS, Sigma). CVB3 was titrated by 50% tissue culture infective dose (TCID_50_) assay on HeLa cell monolayers according to the method of Reed and Muench (5). Acinar cell line 266-6 and pancreatic ductal adenocarcinoma (PDAC) cell line SW1990, gifts from Prof. H. Liu (IBMS, Soochow University, China), were cultured in Dulbecco’s modified Eagle medium (DMEM, Sigma) supplemented with 10% FBS and penicillin and streptomycin (100 U/mL) at 37°C in a 5% CO2 incubator.

### CVB3-induced acute pancreatitis

Six-week-old male C57BL/6 mice were inoculated with 10^3^ PFU CVB3 in 100 μL PBS by intraperitoneal injection (i.p.). The body weight of mice was recorded daily for 14 days following infection. The pancreas was removed on day 0, 3, or 7 post-infection (p.i.) for detection of viral load and histological analysis. Tissues were cut longitudinally, fixed in paraformaldehyde, embedded in paraffin, cut into 5 μm thick sections, and stained with H&E. Images were taken randomly using a TE2000-S microscope (Nikon). Inflammation and edema/necrosis scores were quantified using semiquantitative scoring criteria: 1 means 25% of tissue damage; 2 indicates 25%–50% tissue involvement; and 3 indicates that 50% tissue damage.

### Plaque assay

Pancreas was weighed and homogenized in DMEM containing 2% FBS. After centrifugation at 300 × *g* for 10 min, supernatants were titrated by plaque assay. Briefly, diluted homogenates or cell supernatants were applied onto 95% confluent HeLa cells and incubated for 1 h. After washing twice with PBS, cells were overlaid with a 0.7% agarose-DMEM with 0.2% FBS and cultured at 37°C for 96 h. After removing the agarose, the monolayer was fixed with 4% paraformaldehyde for 1 h and then stained with 0.1% crystal violet for 15 min to visualize and count the plaques.

### Cell transfection and treatment

Cells with 85% confluency were infected with CVB3 at a multiplicity of infection (MOI) of 1, 5, or 10 for 1 h. After washing with PBS, cells were maintained for 12 ~ 24 h. In experiments where CA074Me (Cathepsin B Inhibitor) (TargetMol T3420) was used, cells were treated with 10 µM CA074-Me dissolved in DMSO (Sigma) for 12 h after CVB3 infection (MOI of 1). For CTSB overexpression, cells were transfected with 1 µg pCTSB plasmid or pCDH vector plasmid for 24 h before infection with CVB3. For CTSB knockdown, cells were transfected with siRNA-CTSB using X-tremeGENE siRNA reagent (Roche) for 36 h before CVB3 infection.

### Subcellular fractionation

Cells were collected by centrifugation at 2000 × *g* and washed with ice-cold PBS. The cells were suspended in ice-cold fractionation buffer (20 mM HEPES [pH 7.4], 250 mM sucrose, 10 mM KCl, 1.5 mM MgCl2, 1 mM EDTA, 1 mM EGTA) containing protease and phosphatase inhibitors and passed through a 25 G needle 20 times to homogenize the cell suspension. The lysosome fraction was obtained by lysosome isolation kit (Bestbio, bb31452). Briefly, cells were homogenized with lysis buffer and then centrifuged at 4°C, 20,000 × *g* for 20 min to obtain the supernatant as cytoplasmic fraction. The resulting precipitates were resuspended with lysosome extraction reagent and centrifuged at 30,000 × *g* for 30 min to collect lysosomal fractions, which were suspended in RIPA lysis buffer (50 mM Tris HCl [pH 8], 150 mM NaCl, 1 mM EGTA, 1% NP-40, 0.1% SDS, 0.5% sodium deoxycholate, 5% glycerol) containing protease and phosphatase inhibitors.

### Western blot analysis

The cell pellet and pancreas tissue were lysed with RIPA buffer for 20 min on ice to obtain lysates and then centrifuged at 12,000 rpm for 15 min at 4°C. The cell pellet was resuspended in lysis buffer and incubated on ice for 10 min. The extracted proteins were separated using SDS-PAGE and subsequently blotted onto PVDF membrane (Amersham). The membranes were blocked with 5% non-fat dry milk in Tris-buffered saline containing 0.2% Tween 20 (TBST) for 1 h at room temperature and then incubated with anti-VP1 (Mediagnost; M47), anti-Tubulin (CST, D3U1W), anti-CTSB (CST, D1C7Y), anti-CTSD (CST, E5V4H), anti-LAMP1 (CST, C54H11), anti-GAPDH (CST, D16H11), anti-Annexin A1 (abcam, ab214486), anti-Calnexin (abcam, ab22595), anti-CD9 (ABclonal, A19027), anti-CD63 (ABclonal, A19023), or anti-ALIX (ABclonal, A25326) in 1% non-fat milk-TBST overnight at 4℃. Densitometric measurements were performed using Image J software (version 2.5) and were normalized to loading controls.

### Quantitative real-time PCR (qPCR)

Pancreatic tissues (20 mg) or cells were treated with 1 mL of RNAiso Reagent (AG RNAex Pro RNA, AGbio). Total RNA was extracted using TRIzol RNA isolation reagent (Thermo Fisher Scientific). Semiquantitative RT-PCR analyses with ransStart Green qPCR SuperMix UDG kit (Transgen, China) were conducted on 5 µg of total RNAs on a LightCycler 480 Real-Time PCR System (Roche, Basel, Switzerland) using the specific sets of primers (Table 1, Beijing Tsingke Biotech). The relative abundance of specific mRNA was normalized to that of GAPDH. Relative quantification was determined using the 2−ΔΔCt method.

**TABLE 1:** Primers in experiments in cells

Primer name	Sequence (5′–3’)	Tm
CTSA	F: CAGGCTTTGGTCTTCTCTCCA	
	R: TCACGCATTCCAGGTCTTTG	
CTSB	F: TACCTTCGAGGTACTGGTCCCT	60°C
	R: GGTGGAGAAAGTCCAGCAACTG	
CTSC	F: CAACTGCACCTACCCTGATCT	59°C
	R: TAAAATGCCCGGAATTGCCCA	
CTSD	F: TGCTCAAGAACTACATGGACGC	59°C
	R: CGAAGACGACTGTGAAGCACT	
CTSE	F: GACATCAGTCCCTTCGGAAGA	58°C
	R: AGGGGTTCATTGACACTCGAATA	
CTSF	F: CCCTGGAAGCCACACTAGAG	60°C
	R: GGGCTACAGTCCCTCCTCAG	
CTSG	F: AGGGTTTCTGGTGCGAGAAG	59°C
	R: GTTCTGCGGATTGTAATCAGGAT	
CTSH	F: ACCGTGAACGCCATAGAAAAG	60°C
	R: TGAGCAATTCTGAGGCTCTGA	
CTSJ	F: TGTTAATCCTGTGCTTTGGAGTG	59°C
	R: CTTCCCCAGACTATTCTCCTTGT	
CTSK	F: GAAGAAGACTCACCAGAAGCAG	59°C
	R: TCCAGGTTATGGGCAGAGATT	
CTSL	F: ATCAAACCTTTAGTGCAGAGTGG	60°C
	R: CTGTATTCCCCGTTGTGTAGC	
CTSM	F: ACACAGGGCAGATGTAATTCTTG	59°C
	R: TCCATCACATAGTGCAATGCAA	
CTSO	F: CAGCGTGGTGAGTGCCATAG	60°C
	R: ACCGAGGCAGCCAGAATTATTA	
CTSQ	F: GTGTTTCAGCATTTGATCCCAGT	58°C
	R: GTCAGCAAACCCATTTAATCCCA	
CTSR	F: ACAGCCTGGGTAAGAATGGC	60°C
	R: CCTCTCTGTGAGTCCAAACTGAA	
CTSS	F: CCATTGGGATCTCTGGAAGAAAA	60°C
	R: TCATGCCCACTTGGTAGGTAT	
CTSW	F: TGACTCCCTCCTCACCAAGG	59°C
	R: GCTGGGTTCCAGTAACTCCG	
CTSZ	F: GGCCAGACTTGCTACCATCC	58°C
	R: ACACCGTTCACATTTCTCCAG	
MCOLN1	F: TTGCTCTCTGCCAGCGGTACTA	59°C
	R: GCAGTCAGTAACCACCATCGGA	
ATP6V1H	F: GGAAGTGTCAGATGATCCCCA	60°C
	R: CCGTTTGCCTCGTGGATAAT	
CLCN7	F: TGATCTCCACGTTCACCCTGA	60°C
	R: TCTCCGAGTCAAACCTTCCGA	
LAMP1	F: ACGTTACAGCGTCCAGCTCAT	60°C
	R: TCTTTGGAGCTCGCATTGG	
Cystatin C	F: AAGCCAGCAACGACATGTACC	58°C
	R: GATGTGGCTGGTCATGGAAGG	
GAPDH	F: GGTGGTCTCCTCTGACTTCAACA	59°C
	R: GTTGCTGTAGCCAAATTCGTTGT	
IL-1β	F: GCAACTGTTCCTGAACTCAACT	59°C
	R: ATCTTTTGGGGTCCGTCAACT	
IL-6	F: TGGGGCTCTTCAAAAGCTCC	58°C
	R: AGGAACTATCACCGGATCTTCAA	
IL-17a	F: TTTAACTCCCTTGGCGCAAAA	60°C
	R: CTTTCCCTCCGCATTGACAC	
TNF-α	F: CCCTCACACTCAGATCATCTTCT	59°C
	R: GCTACGACGTGGGCTACAG	
CVB3	F: ATCAAGTTGCGTGCTGTG	59°C
	R: TGCGAAGTGAAAGGAGTGT	

### Cathepsin B activity

According to the manufacturer’s protocol, a total of 50 µL of samples and 50 µL of reaction buffer were dispensed into black 96-well plates. To each well, 2 µL of CTSB substrate (Ac-RR-AFC; final concentration 200 µM) was added and incubated for 1 h at 37°C. Fluorescence was measured using a microplate reader with excitation and emission wavelengths set to 400 nm and 505 nm, respectively.

### Exosome purification

Exosome isolation medium was removed from cells and subjected to 3,000 × *g* centrifugation for 15 min to remove cell debris, then transferred to a new tube. Exosome was purified from cell culture supernatant using Isolation Reagent (Applygen Technologies Inc.), as described previously. Briefly, after filtration through 0.22 µm membrane, exosome purification reagent was added into sample at a 1:5 dilution and incubated at 4°C for 12 h. After 1,500 × *g* centrifugation for 15 min, exosomes were pelleted and subjected to 1,500 × *g* centrifugation for 5 min. Exosomes were resuspended with 100 µL PBS or lysed in RIPA buffer. The virus titer was determined using the plaque assay method.

### Immunofluorescent staining

266-6 cells, cultured on NuncChamber Slide (Nunc, Roskilde, Denmark), were infected with eGFP-CVB3 at a MOI of 1. Twelve hours post-infection, cells were processed for fluorescence microscopy. Briefly, cells were washed with PBS, fixed with 4% paraformaldehyde, and permeabilized with 0.5% Triton X-100. After blocking with 10% goat serum for 30 min, cells were incubated with anti-CTSB (Abcam, USA) for 1 h. For evaluating lysosomal CTSB leakage, 12 hpi, cells were stained with anti-CTSB and anti-LAMP1 (Santa Cruz Biotechnology) for 1 h. Following incubation with an FITC- or PE-labeled goat-anti-mouse antibody (Santa Cruz, USA) for 30 min in the dark, nuclei were counterstained with DAPI for nuclear visualization. Fluorescence images were captured with a confocal microscope (Olympus Corporate, FLUOVIEW FV3000).

### *In vivo* CTSB overexpression or CTSB inhibitor treatment

Mice were injected with 1.0 mL reagent containing 50 µg of mouse p-CTSB plasmid or vector plasmid using *in vivo*-JetPEITM–Gal transfection agent (Polyplus-transfection, USA) via intravenous injection according to the manufacturer’s instruction. Mice received 3 doses of pCTSB at −1, 0, and 1 dpi. For CTSB inhibitor treatment, mice were intraperitoneally (i.p.) administered CA074Me (10 mg/kg) at 0, 1, and 2 dpi with 10³ PFU of CVB3 on day 0. Sham control mice received an equivalent volume of vehicle via the same route. Pancreatic tissues were harvested at 3 or 7 dpi for viral titer quantification and histopathological analysis.

### Statistical analysis

Multiple group comparisons were performed by one-way ANOVA, followed by Bonferroni post hoc tests (StatPlus). Results are presented as mean ± SD. A *P* value < 0.05 was considered statistically significant. The survival curves were plotted using Kaplan-Meier methods, and significance was determined using the log-rank test.

## RESULTS

### CTSB is hyperactivated in CVB3-induced AP

**Fig 1:**
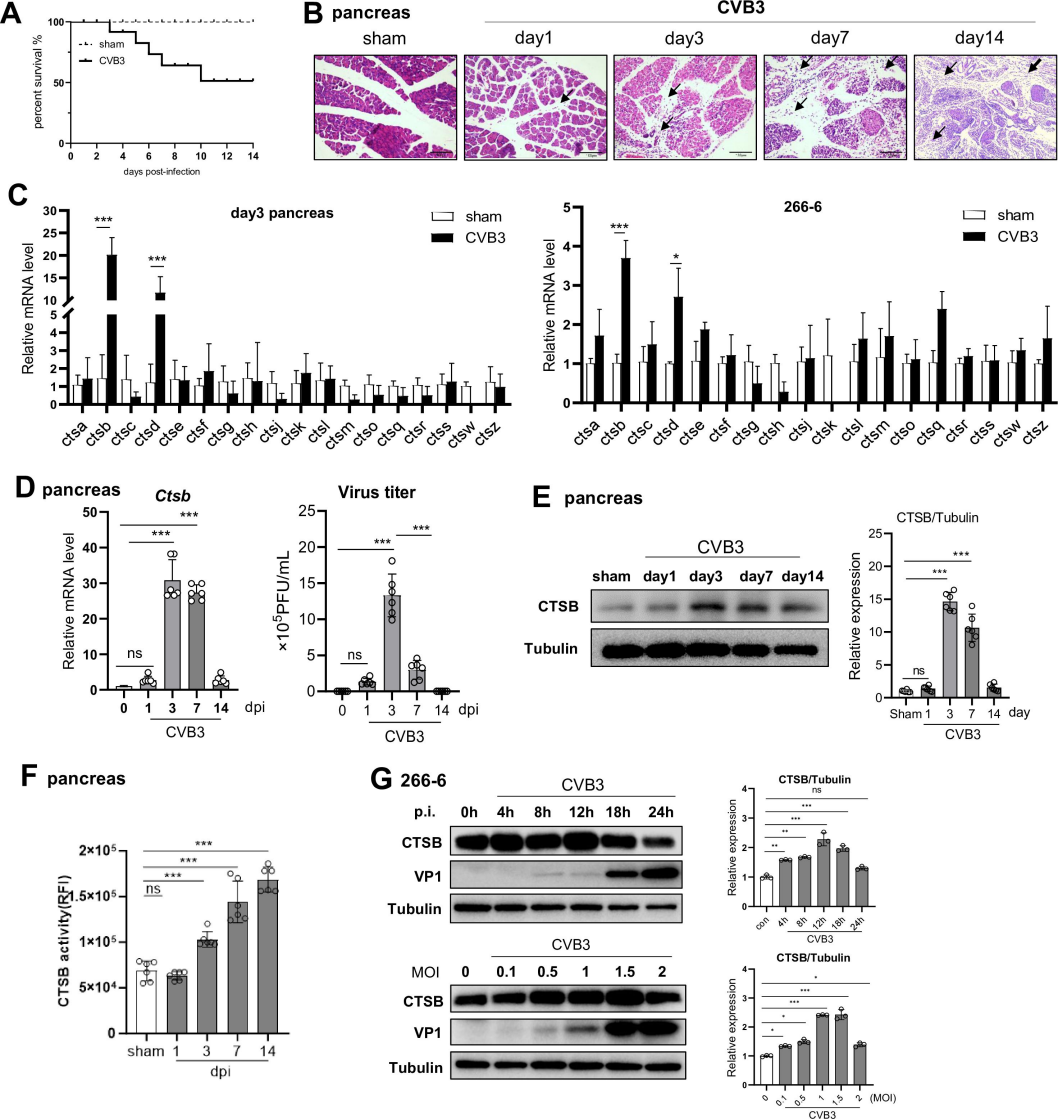
CVB3 upregulates the expression and activity of CTSB. C57BL/6 mice were i.p. injected with 10^3^ pfu CVB3. (**A**) Survival curve of mice infected with CVB3. (**B**) Representative haematoxylin–eosin (H&E)-stained pancreas sections from infected mice. Arrows indicated lymphocyte infiltration. Scale bar: 100 µm. (**C**) Real-time qPCR analysis of mRNA levels of cathepsin family members in day 3 pancreas of CVB3-infected mice or murine 266-6 acinar cells. (**D**) Real-time qPCR detection of CTSB mRNA levels and plaque-forming assay of CVB3 titers in infected pancreas. (**E**) Immunoblotting analysis of CTSB protein using total lysates from pancreas of mice. (**F**) The CTSB activity of infected pancreas was analyzed. (**G**) Immunoblotting analysis of CTSB in 266-6 acinar cells infected with increased doses of CVB3 (MOI = 0.1–2) or maintained for 0–24 h. Densitometric analysis of CTSB normalized to tubulin is shown as fold change. Data from panels **C–G** are represented as mean ± SEM of averages from three independent experiments. ns, no significance. *P < 0.05, **P < 0.01, ***P < 0.001.

To investigate the role of CTSB in viral-induced AP, we employed an experimental model of CVB3-induced AP in which male C57BL/6 mice were i.p. injected with 10^3^ PFU of CVB3. Within the 14-day period, mice experienced 50% mortality (Fig. 1A) and developed severe pancreatic necrosis and inflammatory injury, which began on day 3 and persisted until day 14 post-infection (p.i.) (Fig. 1B). To identify the most upregulated cathepsin in the pancreas during acute CVB3 infection, we screened the mRNA levels of 18 cathepsin family members in day 3 pancreas using real-time qPCR. At 3 dpi, pancreas tissue from infected mice showed a significant increase in mRNA expression of *Ctsb* and *Ctsd*, with their level rising approximately 20-fold and 10-fold, respectively (Fig. 1C), compared to their background expressions. Then, the time course of CTSB during viral AP was determined. The pancreatic *Ctsb* mRNA and protein expression increased starting at day 1, peaked at day 3 p.i., and declined by day 14 p.i., as shown by qPCR and Western blotting analysis (Fig. 1D and E). Notably, the activity of CTSB increased progressively within 14 days of infection (Fig. 1F). Analysis of CTSB levels in CVB3-infected murine acinar cell line 266-6 revealed a time- and dose-dependent increase in CTSB protein expression post-infection (p.i.) (Fig. 1G). Taken together, pancreatic CTSB was elevated and hyperactivated at early viral infection.

### CVB3 infection causes decreased lysosomes, lysosomal membrane permeabilization (LMP), and CTSB relocalization

**Fig 2:**
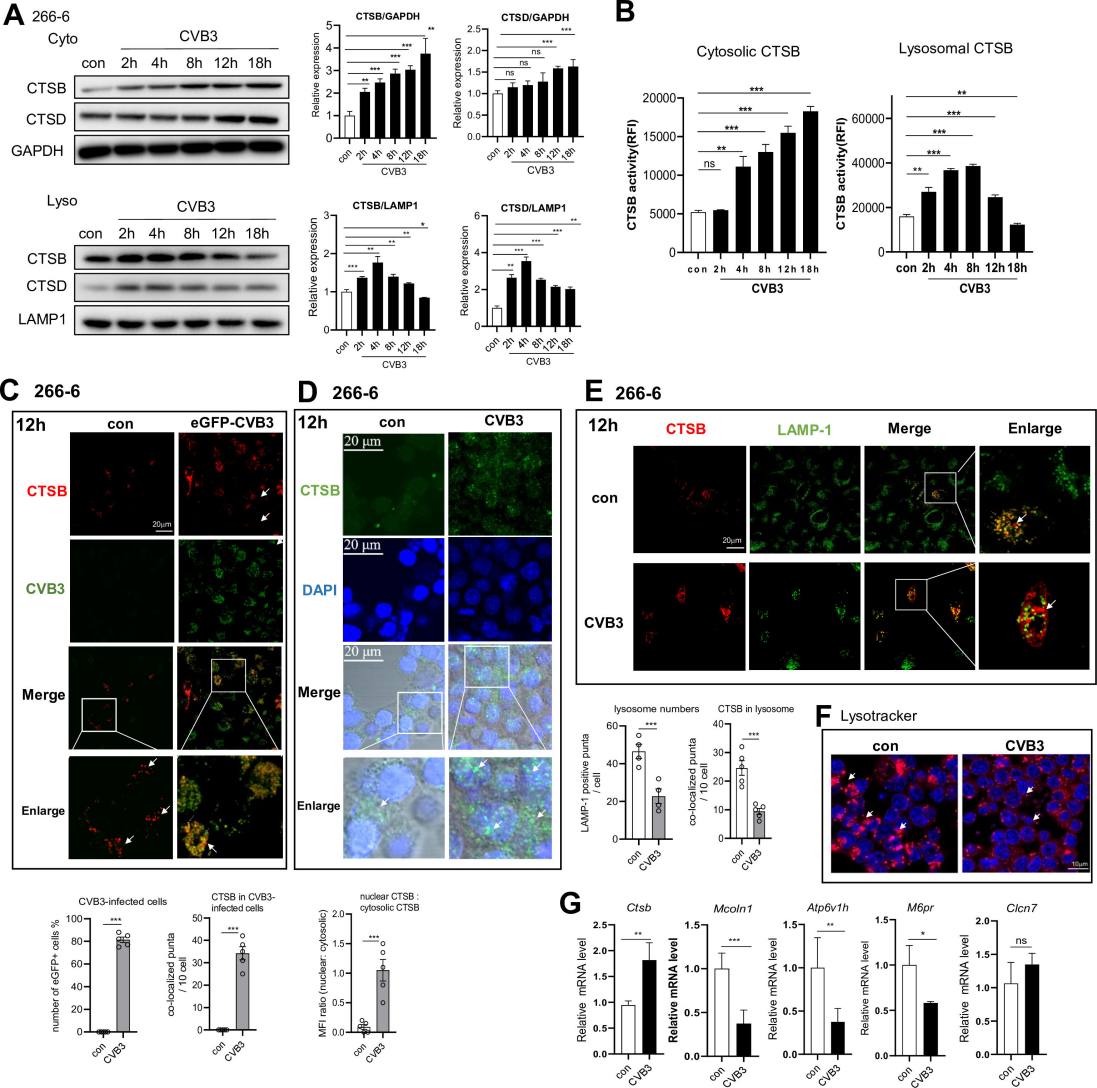
CVB3 infection decreases lysosome numbers, induces lysosomal membrane permeabilization, and CTSB relocalization in acinar cells. (**A**) Immunoblotting analysis of CTSB and CTSD proteins in lysosomal and cytoplasmic fractions of 266-6 cells infected with CVB3 (MOI = 1) for 1 h, then maintained for 12 h. Densitometric analysis of CTSB/CTSD normalized to tubulin or LAMP1 is presented as fold changes compared with control. (**B**) The CTSB activity of cytosolic fractions and lysosomal fractions of infected 266-6 cells. (**C**) Representative fluorescent microscopy images of control and eGFP-CVB3-infected (MOI = 1, for 12 h) cells marked with green (GFP-CVB3) and labeled against CTSB (red). Arrowheads represent colocalized puncta of CTSB with GFP + virus. Arrows indicate red-only CTSB puncta. Scale bar: 20 µm. Numbers of GFP + cells and CTSB/GFP overlay puncta per cell in each group were quantified. (**D**) Fluorescent microscopy images of control and CVB3-infected (MOI = 1, for 12 h) cells marked with DAPI (nucleus) and labeled against CTSB (green). Arrows indicate CTSB puncta. Scale bar: 10 µm. The MFI ratios of nuclear (green) to cytosolic (green) CTSB are presented. (**E**) Representative fluorescent microscopy images of control and CVB3-infected (MOI = 1, 12 h) cells labeled against CTSB (red) and lysosome (LAMP1, green). Arrowheads represent colocalized puncta of CTSB with LAMP1. Arrows indicate red-only CTSB puncta. Scale bar: 20 µm. The numbers of LAMP1 puncta and CTSB/LAMP1 overlay puncta per cell in each group were quantified. (**F**) Fluorescent microscopy images of control and CVB3-infected cells (MOI = 1, for 12 h) labeled with Lysotracker Red. Arrow indicates Lysotracker Red positive staining. Scale bars: 10 µm. (**G**) Real-time qPCR of mRNA levels of lysosomal biogenesis genes in CVB3-infected 266-6 cells. Data from panels **A–E and G** represent as mean ± SEM of averages from three independent experiments. *P < 0.05, **P < 0.01, ***P < 0.001. ns, no significance.

To see whether CVB3 induces lysosomal damage and translocation of cathepsins, 266-6 cells were infected with CVB3 (MOI = 1) for 1 h, maintained for 12 h, and then fractionated into the cytosolic and lysosomal lysates. CTSB and CTSD protein levels in different fractions were assessed by immunoblotting. Notably, lysosomal CTSB level decreased from 8 hpi, while cytoplasmic CTSB expression started to increase from 8 h and maintained a high level until 18 h, indicating leakage of CTSB from lysosome into the cytoplasm, a marker of LMP (Fig. 2A). Accordingly, cytoplasmic CTSB activity increased progressively from 2 to 18 hpi (Fig. 2B).

To further clarify the impact of CVB3 infection on the abundance and subcellular location of CTSB, 266-6 cells were infected with eGFP-CVB3 for 12 h and stained for CTSB. 12 hpi, when over 70% of acinar cells are infected and viral particles are produced, CTSB (red) abundance was markedly increased compared to its low expression in uninfected cells, with most upregulated CTSB localized in CVB3 + cells (Fig. 2C). LMP is often associated with a decrease in lysosomal-associated membrane protein 1 (LAMP1) and the release of lysosomal cathepsins into the cytoplasm (31). The subcellular location of CTSB was determined by confocal analysis of 266-6 cells 12 hpi labeled with CTSB and LAMP-1. Confocal imaging of the lysosomal marker, LAMP1, and CTSB in virus-infected cells showed that the LAMP-1^+^ lysosome numbers were markedly decreased, indicating compromised lysosomal membrane integrity. Moreover, although the CTSB puncta (red) increased after infection, the LAMP1-CTSB colocalization (yellow puncta) was significantly reduced in infected cells, compared with high colocalization in control cells (Fig. 2E), indicating that most of elevated cytosolic CTSB is extra-lysosomal at 12 hpi.

The subcellular location of CTSB was also determined by confocal analysis of cells probed against CTSB (green) and stained with DAPI. As shown in Fig. 2D, CTSB level is low and distributed near the nucleus in control cells, and no nuclear localization was visible. In contrast, at 12 hpi, abundant CTSB proteins were more diffusely distributed in the cytoplasm, with some CTSB puncta (green) observed in the nucleus, as reflected by the ~10-fold shift in the nuclear/cytosolic ratio of CTSB MFI. It indicates that CVB3 infection triggers CTSB nuclear translocation.

Immunofluorescent analysis using Lysotracker indicated a significant decrease in lysosome acidity in infected acinar cells, indicating lysosome acidification impairment and instability (Fig. 2F). qPCR analysis revealed that CVB3 decreased RNA expressions of lysosomal biogenesis genes (*Lamp-1*, *Mcoln1*, *Atp6v1h*, and *M6pr*) in 266-6 cells 12 hpi (Fig. 2G), indicating reduced lysosomal biogenesis and repair. These results suggest that CVB3 induces lysosome instability, LMP, and CTSB cytosolic translocation by reducing lysosome integrity through decreased LAMP1 expression.

### CTSB increases viral replication and virion release in pancreatic acinar cells

**Fig 3:**
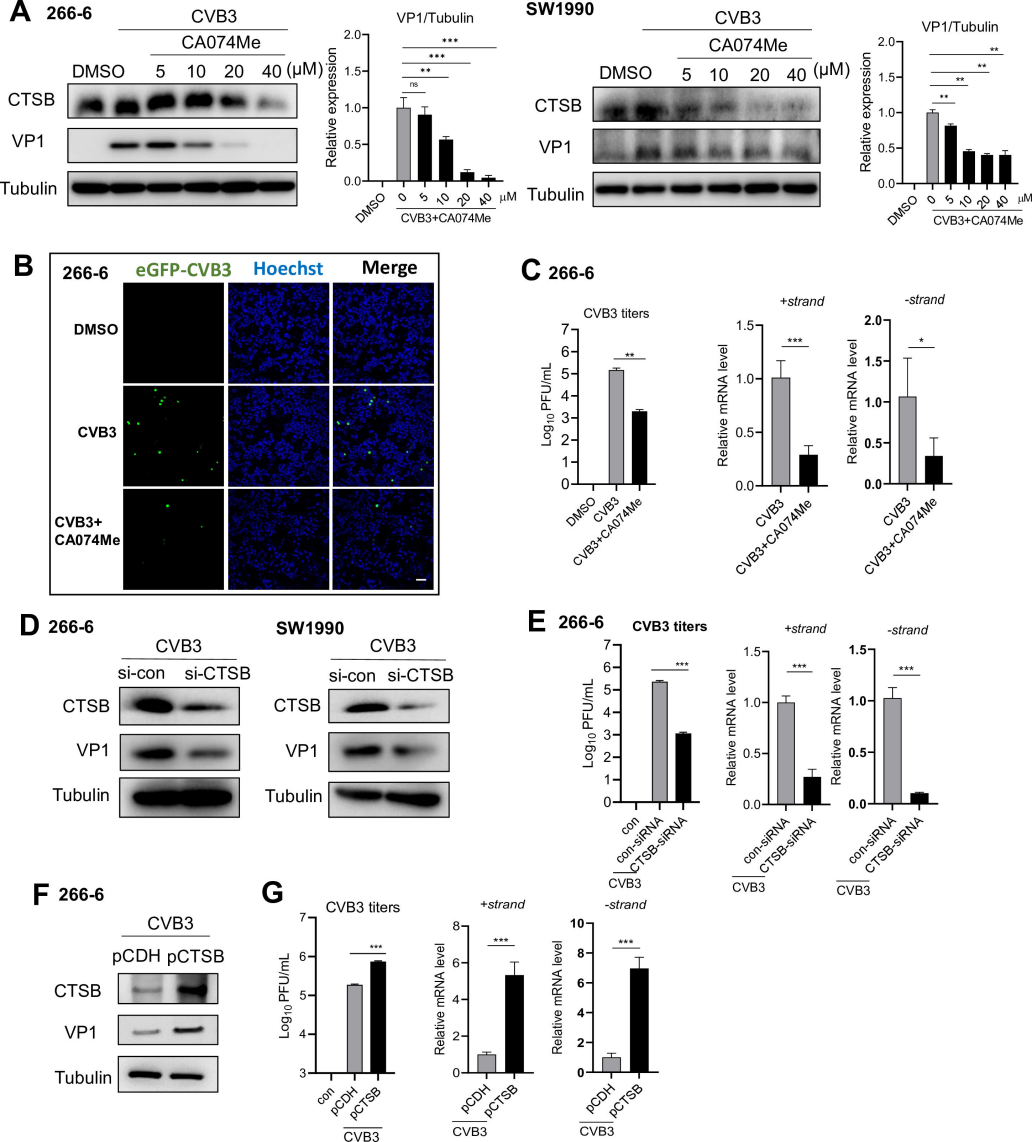
CTSB increases viral replication and virion release in pancreatic acinar cells. (**A**) Immunoblotting analysis of CTSB and VP1 proteins in 266-6 cells and SW1990 cells infected with CVB3 (MOI = 1) for 1 h, and treated with CTSB inhibitor, CA074Me for 12 h. Densitometric analysis of CTSB normalized to tubulin is shown as fold change. (**B**) Confocal microscopy of 266-6 cells infected with eGFP-CVB at MOI = 1 for 1 h and treated with 20 µM CA074Me for 12 h. Scale bars represent 100 µm. (**C**) Quantification of CVB3 titer in CA074Me-treated 266-6 cell culture supernatant by plaque-forming assay or mRNA levels by real-time qPCR. (**D**) Immunoblotting analysis of CTSB and VP1 proteins in 266-6 and SW1990 cells pre-treated with siRNA-CTSB for 36 h, then infected with CVB3 (MOI = 1) for 12 h. (**E**) Plaque-forming assay of CVB3 titer in cell culture or real-time qPCR analysis of CVB3 mRNA levels in 266-6 cells. (**F**) Immunoblotting analysis of CTSB and VP1 proteins in 266-6 cells pre-transfected with pCDH or pCTSB plasmid for 36 h, then infected with CVB3 (MOI = 1) for 1 h, maintained for 12 h. (**G**) CVB3 titer in culture supernatant and viral mRNA expression were determined by plaque forming assay and real-time qPCR. Data from panels **C, E, and G** were shown as mean ± SEM of averages from three independent experiments. *P < 0.05, **P < 0.01, ***P < 0.001.

To confirm the pro-viral role of CTSB in the pancreas, acinar cell 266-6 and pancreatic ductal adenocarcinoma (PDAC) cell line SW1990 cells were analyzed for viral level upon treatment with specific CTSB inhibitor CA074Me (5–40 μM) for 12 h. CA074Me dose-dependently reduced VP1 expression in both cells (Fig. 3A). Immunofluorescence imaging revealed that 12 h after eGFP-CVB3 (MOI = 1) infection, CA074Me (20 µM) treatment led to a marked reduction of eGFP + cells compared to DMSO treatment (Fig. 3B). Plaque assay on the cell supernatant and qPCR assay revealed a substantial reduction in extracellular viral titer and viral mRNA expression after CA074Me treatment (Fig. 3C). Using small interfering RNAs targeting CTSB (si-CTSB) to reduce CTSB expression (Fig. 3D) significantly decreased CVB3 VP1 protein (Fig. 3D), extracellular viral titer, and viral mRNA levels (Fig. 3E). While overexpression of CTSB (Fig. 3F) led to an increase in extracellular viral titer and viral RNA expression (Fig. 3G), these data demonstrate that CTSB increases CVB3 replication and virion release in pancreatic cells.

### Inhibition of CTSB blocks exosome release of CVB3 via rescuing lysosomal integrity and function

**Fig 4:**
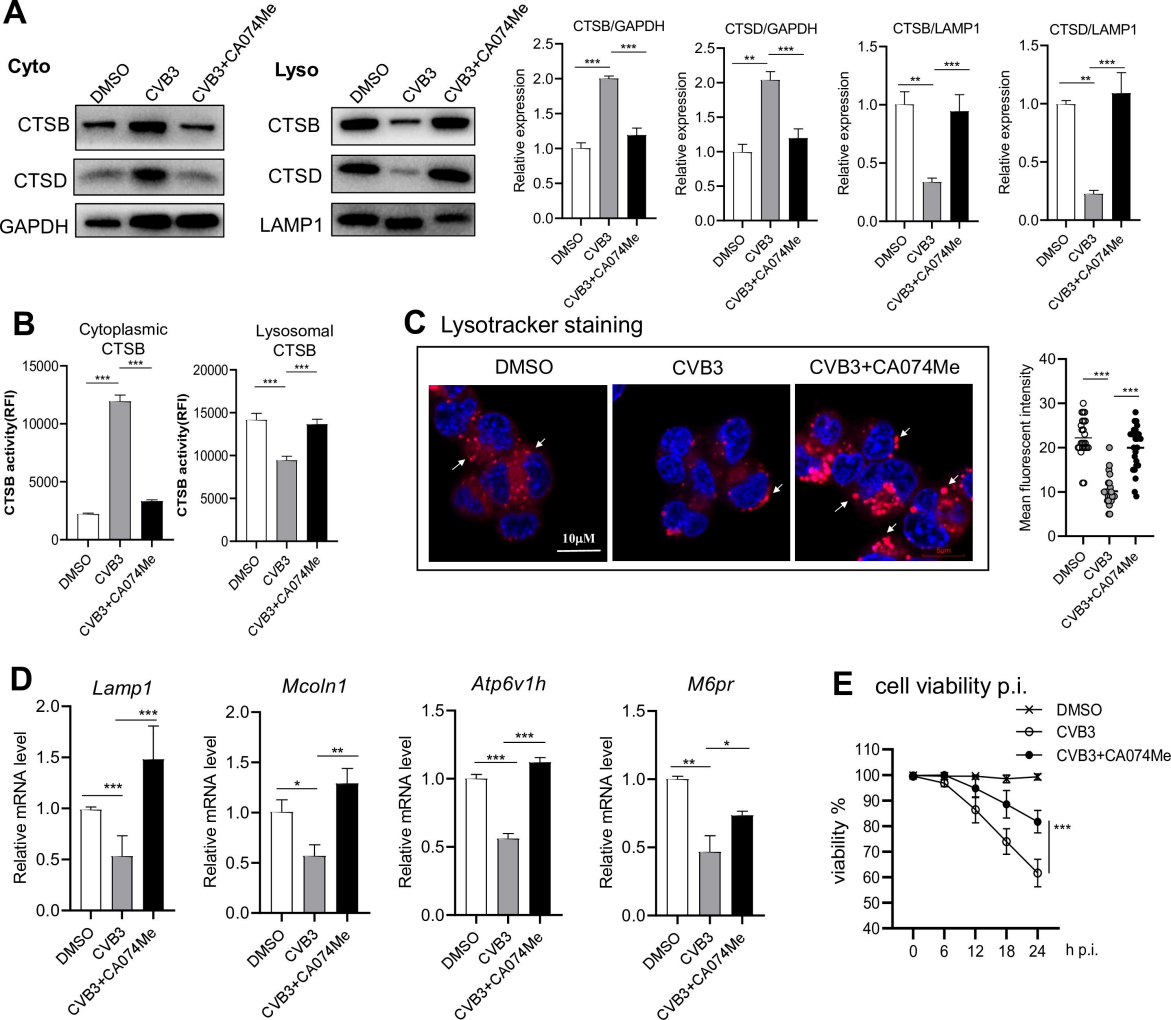
Inhibition of CTSB blocks exosome release of CVB3 via rescuing lysosome integrity and function after infection. 266-6 cells were infected with CVB3 (MOI = 1) for 1 h, washed, and then treated with CA074Me (20 µM) for 12 h. (**A**) Immunoblotting analysis of CTSB and CTSD in cell lysosomal and cytoplasmic fractions lysates. Densitometric analysis of CTSB/CTSD normalized to GAPDH or LAMP1 is shown as fold changes compared with control. (**B**) CTSB activity of CA074Me-treated 266-6 cells. (**C**) Fluorescent microscopy images of control and CA074Me-treated cells after CVB3 infection (MOI = 1, for 12 h) labeled with Lysotracker Red (LTR). Mean fluorescent intensity (MFI) of cells was quantified from *n* = 25 cells per section. Arrow indicates Lysotracker Red positive staining. Scale bars: 10 µm. (**D**) mRNA expressions of *ctsb*, *Mcoln1*, *Atp6v1h*, and *M6pr* in CA074Me-treated cells were analyzed by real-time PCR. (**E**) CCK-8 assay of cell viability 12 h after CA074Me treatment. Data were expressed as mean ± SEM of averages from three independent experiments. *P < 0.05, **P < 0.01, ***P < 0.001.

To ascertain the effect of CTSB hyperactivation on LMP, 266-6 cells were infected with CVB3 for 1 h, then treated with CTSB inhibitor, CA074Me for 12 h, after which the cytoplasmic and lysosomal fractions were subjected to immunoblotting. CTSB inhibitor treatment significantly reduced cytoplasmic but increased lysosomal CTSB/CTSD expression (Fig. 4A), and markedly reduced the cytosolic CTSB activity (Fig. 4B). The Lysotracker Red staining marks the luminal acidity degree necessary for lysosomal protease function. Compared to uninfected cells, virus-infected cells had significantly reduced LTR intensity, indicating impaired lysosome function. Inhibition of CTSB activity largely rescued lysosomal acidity (Fig. 4C). To further evaluate lysosomal biogenesis gene expressions, qPCR analysis revealed that CVB3 infection markedly decreased mRNA expression of *Lamp-1*, *Mcoln1*, *Atp6v1h*, and *M6pr* (Fig. 4D), which were restored by CTSB inhibition, indicating recovery of stability and self-repair function of lysosome. Cell viability was increased by CTSB inhibitor treatment (Fig. 4E). Collectively, these data indicate that inhibition of CTSB rescues lysosome integrity and function.

**Fig 5:**
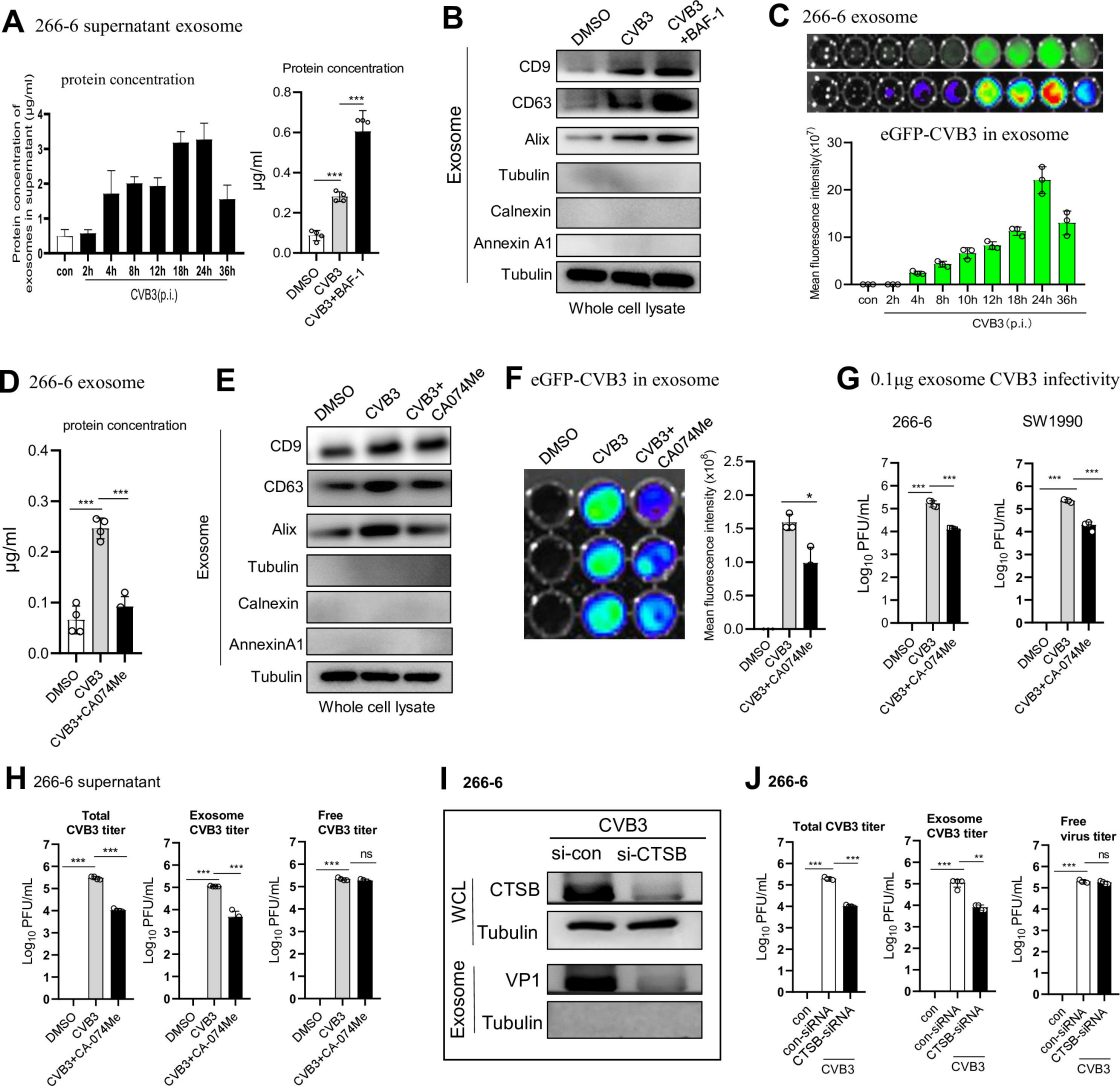
Inhibition of CTSB blocks exosomal release of virus. 266-6 cells were infected with CVB3 (MOI = 1) for 1 h, maintained in the presence of CA074me (20 µM) for 12 h. (**A**) BCA assay analysis of exosomes harvested from CVB3-infected 266-6 cells at indicated times. (**B**) Immunoblotting analysis of exosomal markers (CD9, CD63, and Alix) expression in exosomes purified from supernatant of 266-6 cells infected with CVB3 for 1 h and treated with bafilomycin A1 (20 nM) for 12 h. (**C**) Relative fluorescence units (RFU) detection of GFP + virus in exosomes from 266 to 6 cells infected with GFP-CVB3 (MOI = 1). (**D**) BCA assay of exosomal protein levels harvested from culture supernatant of infected 266-6 cells. (**E**) Western blot detection of exosomal markers CD9, CD63, and Alix expression in exosome lysates. (**F**) Relative fluorescence units (RFU) detection of eGFP-CVB3 progeny in 266-6 cells after CVB3 infection (MOI = 1) for 24 h. (**G**) Viral titers of 266-6 or SW1990 cells infected with 0.1 µg purified exosomes (from infected 266-6 cells treated with CA074Me). (**H, J**) Plaque assay determination of viral titers of exosome lysate from 1 mL supernatant of 266-6 cells treated with CA074Me (**H**), or from cells transfected with con-siRNA or CTSB-siRNA 36 h before CVB3 (MOI = 1) infection (**J**). Total viral titer: viral titer in whole cell culture supernatant; exosome viral titer: free viral titer in the exosome-removed supernatant. (**I**) Western blot analysis of CTSB and VP1 levels in 266-6-exosome and whole cell lysate (WCL). Data from panels** A, C, D, F, G, H, and J** were shown as mean ± SEM of averages from three independent experiments. *P < 0.05, **P < 0.01, ***P < 0.001.

When lysosomal function is impaired, the degradation of endosome/MVB or autophagosome through lysosomes is compromised. This leads to the accumulation of MVB and autophagosome/amphisome, and the release of exosomes (contained in MVB) could be promoted (31). To clarify whether CTSB hyperactivation increases exosome release in acinar cells, exosomes in 266-6 culture supernatant were purified at different infection times. Total protein expression and levels of exosomal markers (CD9, CD63, and Alix), the non-exosomal ER marker (calnexin), and the MV marker (Annexin A1) in exosomes were characterized by immunoblotting. CVB3 infection increased exosomal protein expression in a time-dependent manner (Fig. 5A) and markedly increased CD9, CD63, and Alix exosome marker expressions compared to those in uninfected cells (Fig. 5B). Treating cells with the V-ATPase (acidification) inhibitor bafilomycin A1 (BAF) led to higher exosome production (Fig. 2A and B). Many studies reveal that positive-sense RNA, including Coxsackievirus, exploit the secretory autophagy pathway to exit cells (32). To explore whether CVB3-induced lysosomal dysfunction affects viral release, we infected 266-6 with eGFP-CVB3, and we observed that eGFP^+^ particles in the exosomes were continuously increased from 4 h, peaked at 24 h, and were maintained at 36 hpi (Fig. 5C), showing kinetics similar to that of exosome release.

To examine whether CTSB inhibitor influences exosome release, exosomes were purified from 266 to 6 cell culture supernatant 24 hpi. The increased exosomal protein quantity after infection was largely reduced by CTSB inhibitor (Fig. 5D). Immunoblotting of cell lysates against exosome markers revealed that CVB3 increased exosome production in acinar cell supernatant compared to non-infected cells, which was partially suppressed by CTSB inhibitor (Fig. 5E). To directly show that exosomes contain elevated levels of virions, 266-6 cells were infected with eGFP-CVB3 (MOI = 1) for 1 h and then treated with CA074Me for 24 h, and the viral progeny in exosomes was detected by fluorescent microscopy. The high mean fluorescence intensity (MFI) of exosomal eGFP-CVB3 from infected 266-6 cells was markedly blocked by CA074Me (Fig. 5F). Further, plaque assay on 1 mL culture supernatant-derived exosomes revealed that CVB3 increased exosomal virions release, and CTSB activation mainly increases exosomal virion release, not free virion release (Fig. 5H). Additionally, 0.1 µg purified exosome (from infected 266-6 cells) was examined for infectivity on 266-6 or SW1990 cells, revealing that a high number of infective virions were contained in the exosome, which was reduced by CTSB inhibition (Fig. 5G). It indicates that the antiviral mechanism of CA074Me is primarily mediated through interfering exosome release rather than the virion itself. Downregulating CTSB by transfecting cells with CTSB-siRNA 36 h prior to infection reduced VP1 protein in the exosomes (Fig. 5I). The antiviral effects of CTSB inhibitor or CTSB-siRNA mainly rely on targeting exosome-associated virus, not the free virus (Fig. 5J). Taken together, CTSB increases exosome release of viral particles via disrupting lysosomal integrity and function in acinar cells.

### *In vivo* CTSB overexpression aggravates CVB3 infection and viral AP pathology

**Fig 6:**
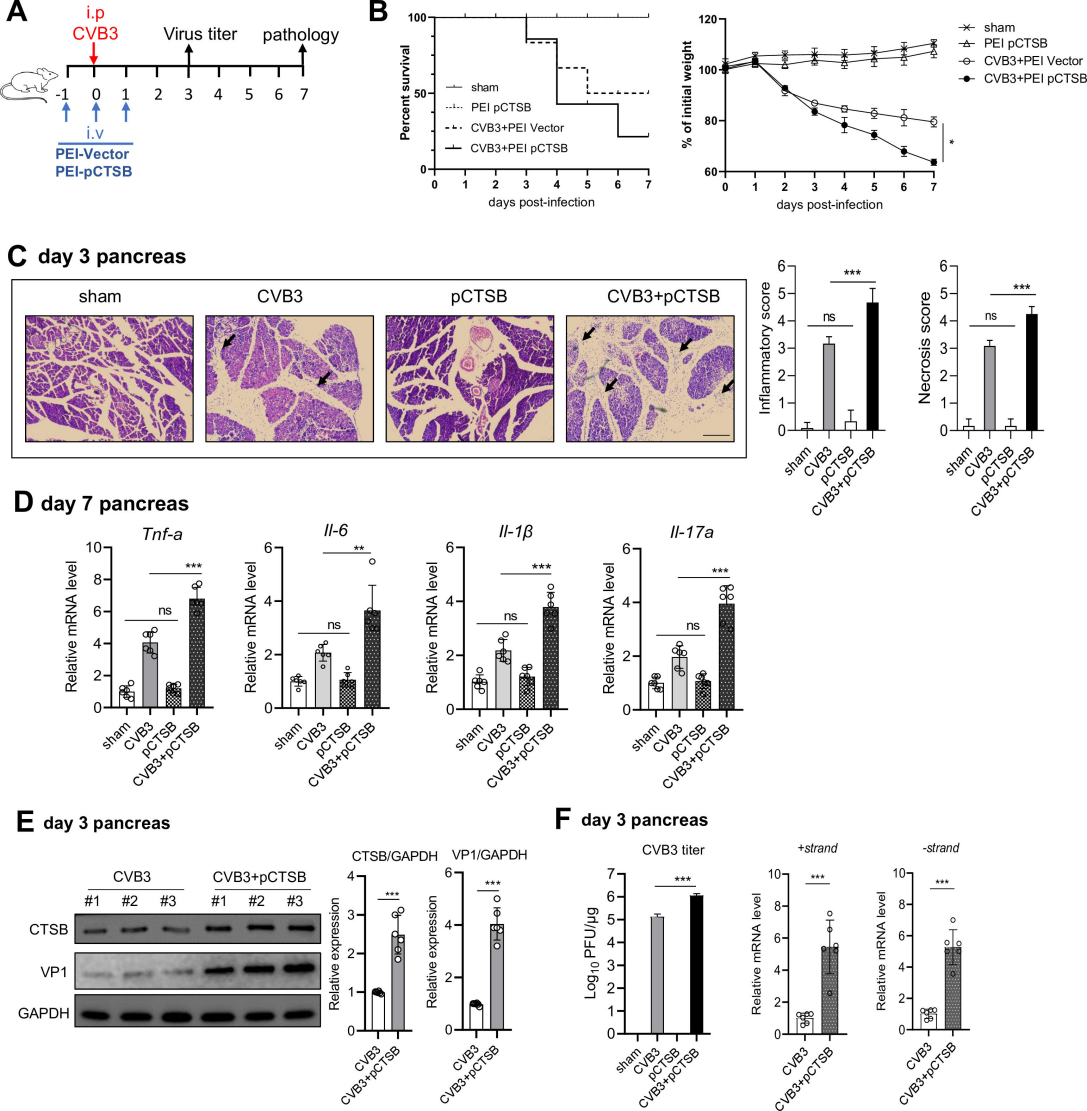
*In vivo* overexpression of CTSB increases CVB3 replication and aggravates AP pathology in mice. (**A**) Mice were i.v. injected 50 µg pCTSB plasmids using *in vivo*-Jet PEI on −1, 0, and 1 dpi. (**B**) Survival curve and weight loss curve in sham, PEI -vector, PEI-pCTSB, and PEI-pCTSB mice were followed by 7 dpi. (**C**) Representative images of H&E staining of pancreatic tissues from infected mice. Arrows indicate lymphocyte infiltration. Scale bar: 100 µm. (**D**) mRNA levels of *Il-6*, *Il-1β*, *Il-17a*, and *Tnf-α* from day 7 pancreas were analyzed by real-time qPCR. (**E**) Immunoblotting analysis of day 3 total pancreas lysates for VP1 expression. (**F**) Viral titer in day 3 pancreas homogenates was determined by plaque assay. Viral mRNA levels were analyzed by real-time PCR. Data from panels** C, D, E, and F** were shown as mean ± SEM of averages from three independent experiments. ns, not significant. *P < 0.05, **P < 0.01, ***P < 0.001.

We next evaluate the effect of CTSB overexpression in mice using an *in vivo*-JetPEITM strategy. Mice were intravenously (i.v.) injected with 50 µg p-CTSB or p-vector on day −1, 0, and 1 dpi using the *in vivo*-Jet PEI reagent (Fig. 6A). Susceptibility to CVB3-AP and viral titer were evaluated within 7 days of infection. CTSB-overexpressing mice exhibited worse disease condition, increased mortality (75 vs 50%, pCTSB vs vector), and increased weight loss by day 7 p.i. (37 vs 19%, pCTSB vs vector, Fig. 6B). Histopathology analysis revealed that CTSB overexpression led to a significantly aggravated acinar cell necrosis and increased immune infiltration in the pancreas at day 3 (Fig. 6C) compared to sham- or p-CTSB-treated mice. Consistent with the pathology, levels of pancreatic inflammatory cytokines (Tnf-α, Il-6, Il-1β, Il-17a) were significantly higher in CTSB-overexpressing mice than in control mice (Fig. 6D). To test whether the above results were due to differences in viral load, pancreatic CVB3 burden was measured. At 3 dpi, peaking time for viral replication, CTSB-overexpressing mice showed increased VP1 protein expression (Fig. 6E), CVB3 mRNA levels, and viral titers (Fig. 6F) in the pancreas compared to sham- or p-CTSB-treated control mice. It indicates that CTSB overexpression promotes viral replication and viral AP pathology *in vivo*.

### Therapeutic administration of pharmaceutical CTSB inhibitor reduces viral infection and AP severity

**Fig 7:**
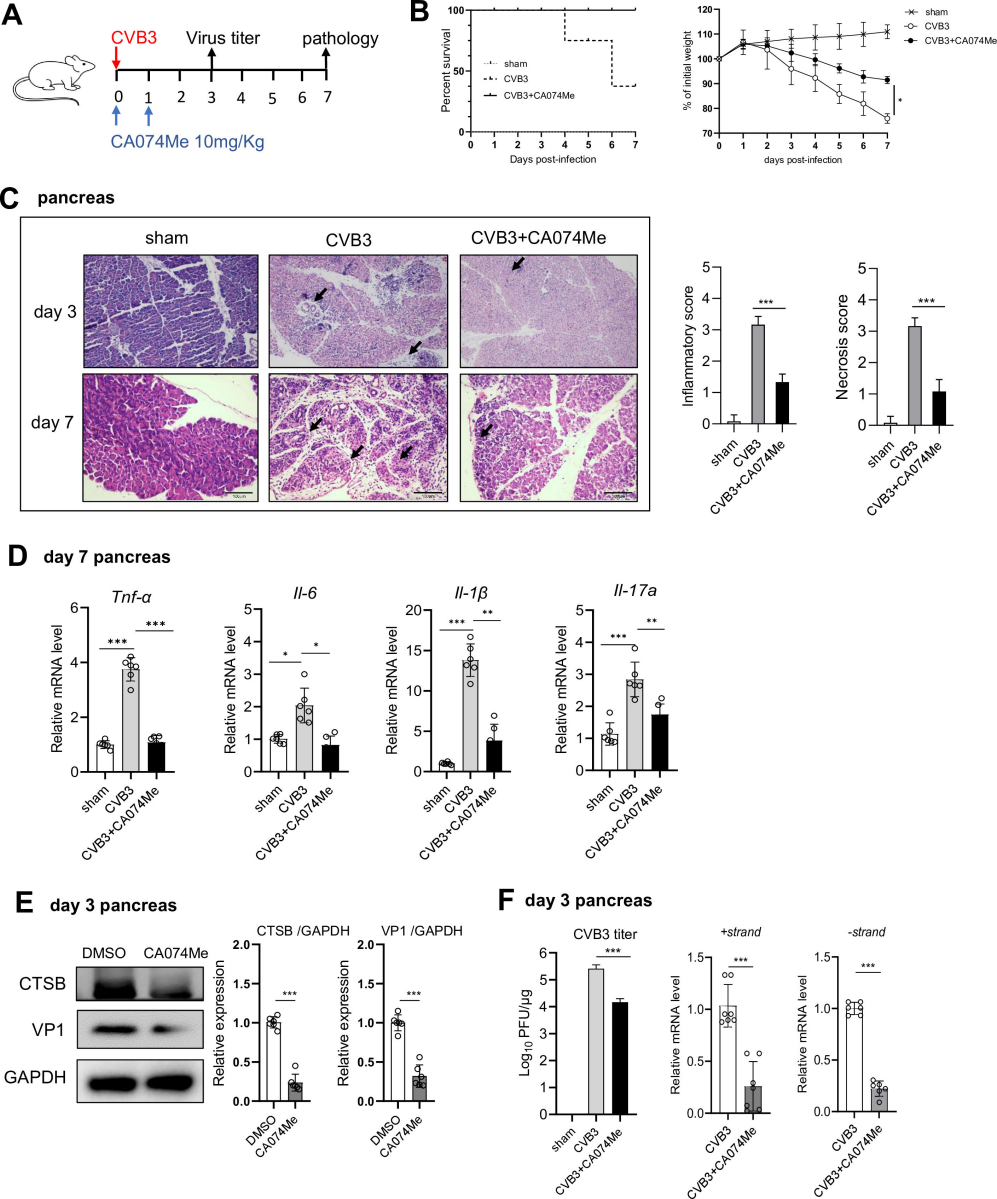
Therapeutic administration of CTSB inhibitor reduces viral infection and AP pathology in mice. (**A**) Schematic map of CA074Me treatment experiment. Mice were i.p. injected with 10.5 mg/Kg CA074Me on day 1 and day 2 after 10^3^ pfu CVB3 infection on day 0. (**B**) Survival curve and weight loss curve of sham, CVB3, and CA074Me-treated mice were followed by 7 dpi. (**C**) Representative images of H&E staining of pancreatic tissues. Arrows indicate lymphocyte infiltration. Scale bar: 100 µm. (**D**) mRNA levels of *Il-6*, *Il-1β*, *Il-17a*, and *Tnf-α* from day 7 mice pancreas were analyzed by real-time qPCR. (**E**) Immunoblotting analysis of day 3 pancreas total lysates for VP1 expression. (**F**) Plaque assay on day 3 pancreas homogenates to determine viral titer. Viral mRNA levels were analyzed by real-time qPCR. Data from panels **C, D, E, and F** were shown as mean ± SEM of averages from three independent experiments. *P < 0.05, **< 0.01, ***P < 0.001.

To evaluate the treating effect of CTSB inhibitor on CVB3-induced AP, mice were i.v. injected with 10 mg/Kg of CA074Me on day 0 and day 1 p.i. (Fig. 7A). By day 7 p.i., CA074Me-treated mice exhibited higher survival rates and weight recovery compared to untreated mice, suggesting improved disease progression (Fig. 7B). CTSB inhibitor significantly reduced acini necrosis and immune infiltration in the pancreas of infected mice on days 3 and 7 p.i. (Fig. 7C). A significant decrease in pancreatic mRNA levels of proinflammatory cytokines (*Tnf-α, Il-6, Il-1β, Il-17a*) was confirmed (Fig. 7D). CTSB inhibition led to marked reduction in VP1 protein, viral titer, and viral mRNA expression in the pancreas of treated mice compared to control mice (Fig. 7E and F). Taken together, targeting CTSB exhibits good treating effect on viral-induced AP by decreasing viral infection and pancreatic inflammatory injury.

## DISCUSSION

The current study tests the effects of CTSB hyperactivation on lysosome integrity and exosome secretion in pancreatic acinar cells using an experimental murine model of CVB3-induced viral AP. We found that CVB3 impairs lysosomal function by decreasing LAMP-1 expression, lysosome biogenesis, and acidification, while markedly upregulating the expression and activity of lysosomal CTSB, as well as its cytosolic translocation. CTSB facilitates exosome release of viral particles through impairing lysosomal integrity and function. Notably, inhibition of CTSB improves lysosomal function and decreases exosome release, thus mitigating pancreatic acini necrosis and inflammation. We highlight the correlation between CTSB hyperactivation, decreased LAMP-1, and exosome secretion, which provide CTSB and exosome as potential targets for the treatment of viral AP.

Known viral pathogens associated with pancreatitis include hepatitis viruses, Coxsackie viruses, and coronaviruses (33, 34). Direct cytopathic effects, trypsinogen activation, and immune infiltration contribute to the pathogenesis of viral AP, but the mechanism is far from illustrated. Apart from trypsin-mediated autodigestion in the early stage of AP, multiple parallel mechanisms, including endoplasmic reticulum stress, autophagy flux impairment, mitochondrial dysfunction, and inflammatory cascade activation in acinar cells, collectively drive the severe systemic inflammatory response and extensive pancreatic damage characteristic of AP (35).

Lysosomal cathepsins (CTSs) comprise a family of serine, aspartic (CTSD), and mainly cysteine (CTSB) proteases, which are important for lysosomal degradative functions (36). Early studies have proposed a critical role of CTSB in intrapancreatic trypsinogen activation and the onset of acute pancreatitis (27, 30). CTSB-dependent conversion of premature trypsinogen into trypsin triggers a subsequent cascade of digestive enzyme activation (elastase, phospholipase A2), leading to pancreatic autodigestion and tissue damage (35). Trypsinogen activation also occurs in pancreatic-infiltrated macrophages, which depends on endocytosis of zymogen-containing vesicles and CTSB activation, leading to macrophage NF-κB activation and systemic inflammation (37). In addition, cytosolic CTSB activates the intrinsic pathway of apoptosis through cleavage of Bid and activation of Bax in acinar cells, leading to necrotic cell death (26). Excessive CTSB activation leads to various forms of cell death, including apoptosis, necrosis, pyroptosis, and ferroptosis (11, 28, 38, 39).

CTSB is a cysteine proteolytic enzyme widely expressed in various cells and mainly located in the lysosomes. In cerulein- or Arg-1-induced experimental AP, activated CTSB protein levels and its activity have been found to be upregulated (27, 28, 38). In a mice model of CVB3-induced viral myocarditis and pressure overload-induced cardiac hypertrophy, CTSB was upregulated in cardiomyocytes and promoted cardiac inflammation and dysfunction through activating the inflammasome or TNF-α/ASK1/JNK apoptotic signaling pathway (11, 29). The current study evaluates the time course of CTSB expression and activity using a CVB3-induced murine AP model. We confirm a significant increase in pancreatic CTSB/CTSD expression both *in vitro* (Fig. 2A through C) and *in vivo* (Fig. 1C, E and F). Interestingly, although CVB3 infection induces overexpression and activation of pancreatic CTSB, it also reduces the expression of lysosomal biogenesis genes (*Lamp-1*, *Mcoln1*, *Atp6v1h*, and *M6pr*), as well as Lamp-1 protein expressions (Fig. 2C through E and 4D) and lysosomal acidity (Fig. 2F and 4C). This suggests a significant lysosome instability and LMP, leading to the translocation of CTSB from lysosome to the cytoplasm and the nucleus (Fig. 2A, C and E). The upregulated CTSB further disrupts lysosomal integrity, whereas CTSB inhibitor restores lysosomal acidity (Fig. 4C). CTSB hyperactivation increases viral titer by enhancing exosome release of viral particles (Fig. 5). Pharmaceutical inhibition of CTSB improves lysosomal function and decreases viral release, eventually leading to mitigated AP pathology (Fig. 4, 5 and 7), demonstrating a detrimental role of CTSB activation in viral AP. Halangk et al. first found that Ctsb-KO mice exhibited partly reduced pancreas damage during cerulein-induced pancreatitis, as assessed by serum amylase activity, pancreas edema, and acinar cell necrosis. However, CTSB deletion had no impact on proinflammatory changes during cerulein-pancreatitis (27). A recent study found that Ctsb-KO mice, which were deprived of intrapancreatic trypsin activity, did not exhibit a mitigated cerulein-pancreatitis (40). Therefore, CTSB activation and function may be versatile in various models of experimental AP. We highlight a pivotal role for hyperactivated and cytosolic translocated CTSB in disrupting lysosome integrity by decreasing the expression of LAMP-1, a key lysosomal membrane protein whose loss of expression is associated with LMP (31). We demonstrate that CVB3 infection significantly reduced lysosome numbers at 12 hpi by decreasing mRNA and LAMP-1 protein expression (Fig. 4D and 2E). Notably, LAMP1-CTSB colocalization was significantly reduced in infected cells compared to the high co-localization in sham cells (Fig. 2E), indicating that the upregulated CTSB proteins are not localized in lysosomes but are distributed in the cytosol. A previous study has demonstrated that cytosolic-translocated CTSB directly degrades LAMP-1 (31). We find that cytosolic CTSB activity was greatly increased (Fig. 2B and 4B), which may accelerate the reduction of LAMP-1, exacerbating lysosome membrane instability.

Lysosomes are membranous organelles that play pivotal roles in macromolecule digestion, signal transduction, autophagy, and cellular homeostasis. Lysosome instability is associated with various pathologies, including cancer, neurodegenerative diseases, inflammatory diseases, and infections (41). Serving as the terminal stations of the endocytic pathway, lysosomes have indispensable roles in the degradation of endogenous and exogenous macromolecules, damaged or superfluous organelles, and pathogens. The autophagy-lysosome pathway is essential for maintaining cellular proteostasis and is associated with viral restriction and viral disease progression. Upon infection, the host initiates the autophagy-lysosome pathway to eliminate damaged organelles, proteins, and exogenous pathogens (42). Impaired autophagy/autophagolysosome has been implicated in experimental and human pancreatitis (43). Positive-strand RNA viruses have employed various mechanisms to antagonize and evade host’s autophagy-lysosome degradation system (32). First, CVB3 exploits endosomal/autophagosomal membrane and remodels them into special structures (closed single-membrane tubules to DMVs and multilamellar structures) for viral RNA synthesis (16). Moreover, upon endocytosis-mediated entry into the cell, instead of accessing endosome–lysosome or autophagy-lysosome degradation pathway, CVB3 and other viruses have evolved diverse mechanisms to avoid interactions with lysosome, thereby evading lysosomal degradation (11, 44). Vesicular stomatitis virus (VSV) nucleocapsid regulates the dynamics of multivesicular endosomes by transferring itself to LBPA-containing intraluminal vesicles (ILVs) and releasing virus into the cytoplasm via fusion with the limiting membrane (45). CVB3 uses the viral protease 2A to cleave p62 and inhibit autophagic degradation of virions (virophagy) (46). HIV-1 Nef interacts with Beclin 1 to sequester TFEB in the cytosol, thus inhibiting maturation of autophagosomes and lysosome biogenesis (47). SARS-CoV-2 virulence factor ORF3a blocks lysosome function by modulating TBC1D5-dependent Rab7 GTPase cycle and impedes the fusion of autophagosomes with lysosomes, causing accumulation of membranous vesicles for replication (48). CVB3 proteinase 3C disrupts host lysosomal function via proteolysing transcription factor EB (TFEB), a master regulator of autophagy and lysosome biogenesis, into a lower-molecular-mass, loss-of-function cleavage fragment, thereby enhancing viral infection (10). During CVB3 infection, fasting/restricted feeding (FR) treatment increased viral RNA levels in multiple organs and accelerated pathology via CTSB-dependent intact autophagy-lysosome pathway, indicating that viral replication and release require functional autophagy and lysosomal pathways (12).

In the current study, we provide new evidence of how CVB3-hyperactivated lysosomal CTSB enhances exosome-mediated viral dissemination through disrupting lysosome integrity and function. Exosomes, a subset of extracellular vesicles generating through the fusion of specific endosomes (MVB) with the plasma membrane, are critical for cell–cell communication, viral dissemination, and cancer metastasis (15, 18). A major source of exosomes is intraluminal vesicles (ILVs) within MVBs. The autophagosome–lysosome system modulates the biogenesis and degradation of exosomes (49). MVBs containing ILVs can either be targeted for degradation in lysosomes or released as exosomes into the extracellular space (49). Lysosomes are essential for the degradation of MVBs, ILVs, and autophagosomes (15). Hypoxia impairs lysosomal degradation by downregulating ATP6V1A expression, leading to the reduced fusion of MVBs with lysosomes and enabling the secretion of ILVs as EVs (50). PTEN facilitates lysosome biogenesis and acidification by dephosphorylating TFEB. PTEN deficiency increases exosome secretion by reducing lysosome-mediated degradation of MVBs (51). Endolysosomal fusion BORC-ARL8-HOPS pathway is a critical determinant of the amount of exosome secretion (52). ER stress activating PERK and IRE1α (UPR) could repress the acidification and catabolic activity of lysosomes, leading to MVB-lysosome fusion block and redirecting MVBs from lysosomal degradation to plasma membrane fusion, resulting in exosome release (53).

Viruses harness the autophagy-lysosome pathway to secrete EVs (exosomes containing viral particles), thereby enhancing infectivity (47). Pathogens, such as enteroviruses, compromise lysosomal membrane integrity by inducing membrane permeabilization, causing cytosolic leakage of proteases and cations, which further induce cell death pathways (54). CVB3 activates TFEB by inactivating mTORC1 signaling, promoting the non-lytic release of CVB3 via a secretory autophagic pathway during the early stages of infection (55). Poliovirus uses phosphatidylserine (PS)-enriched autophagosome vesicles for non-lytic release. Vaccinia virus (VACV) hijacks ESCRT-mediated MVB formation to facilitate virus egress and spread (56). SARS-CoV-2 ORF3a activates the SNARE complex (STX4-SNAP23-VAMP7), inducing fusion of lysosomes with the plasma membrane for viral release in exosomes (48, 57). Viruses exploit these intricate lysosomal-exosomal connections to manipulate incomplete autophagy, enhancing their escape from the exosomal pathway (33). Our findings reveal an intriguing relationship between lysosome inhibition (LMP, biogenesis, and acidification impairment) and exosome secretion in acinar cells during CVB3 infection, which is exacerbated by CTSB. Acinar CTSB hyperactivation facilitates exosome viral secretion and acinar cell necrosis by impairing lysosome integrity and acidification. These data indicate that intact lysosomal integrity and degradation function are critical for blocking exosome release of virions, which is highly efficient for the systemic and receptor-independent infection and dissemination of CVB3 in animals (17). Sustained lysosome dysfunction contributes to chronic disease pathologies.

Our findings reveal a critical role for hyperactivated CTSB-mediated disruption of lysosomal integrity and function in promoting non-lytic viral release and acinar cell necrosis in AP. Pharmaceutical inhibition of CTSB protects mice against viral dissemination and AP pathology by blocking exosome release. Our results suggest targeting CTSB or exosome as alternative therapies for viral AP. Better understanding of CTSB-mediated lysosome disruption and exosome formation mechanisms is essential for developing therapeutic interventions against viral-induced AP.

## Data Availability

The data sets generated and/or analyzed during the current study are available in the figshare repository, accessible via https://doi.org/10.6084/m9.figshare.29364602.v3.
